# Supplementary material for: Neuroprotection of Cholinergic Neurons with a Tau Aggregation Inhibitor and Rivastigmine in an Alzheimer’s-like Tauopathy Mouse Model
Source: Cells. 2024 Apr 6;13(7):642. doi: 10.3390/cells13070642 (PMC11011792; doi:10.3390/cells13070642)
Supplement: Supplementary file 1 [file cells-13-00642-s001.zip › cells-2914578-supplementary.pdf]

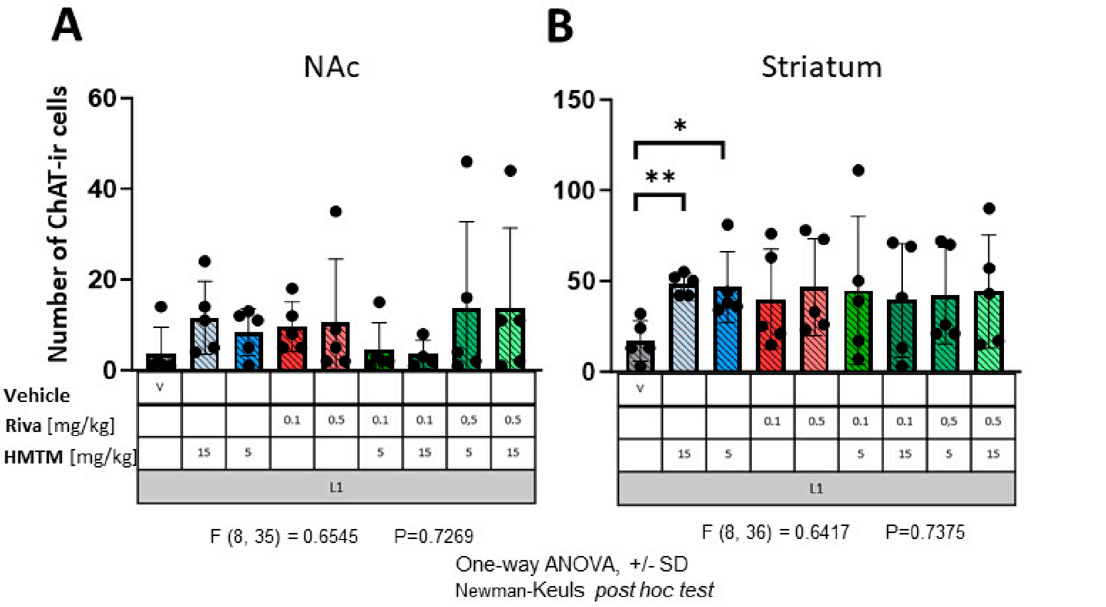

Figure S1. Choline acetyltransferase (ChAT) immunohistochemistry in the basal forebrain in L1 mice. Mean number of ChAT-ir interneurons in **(A)** the nucleus accumbens (NAc) and **(B)** striatum. Values expressed as mean ± S.D. *, p<0.05; **, p<0.01.

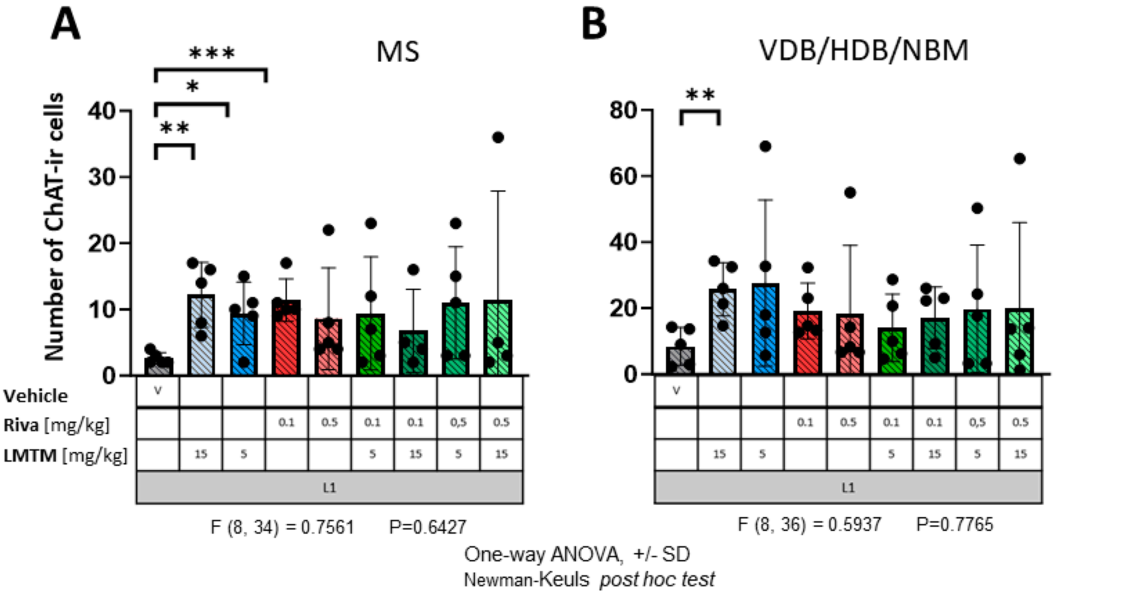

Figure S2. Choline acetyltransferase (ChAT) immunohistochemistry in the basal forebrain in L1 mice. Mean number of ChAT-ir interneurons in **(A)** the medial septum and **(B)** total number of neurons in the vertical (VDB) and horizontal (HDB) limb of the diagonal band of Broca and the magnocellular basal nucleus-substantia innominata (NBM). Values expressed as mean ± S.D. *, p<0.05; **, p<0.01; ***, p<0.001.

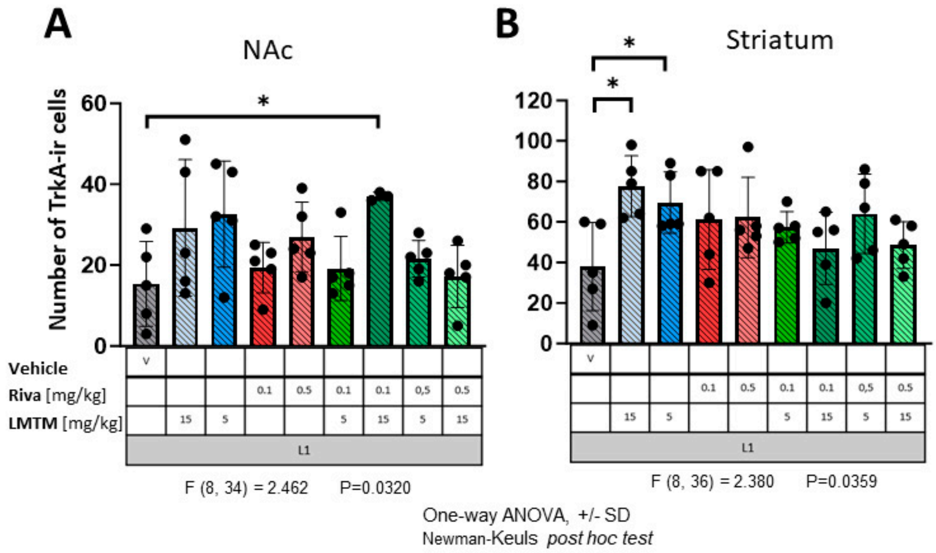

Figure S3. High affinity nerve growth factor receptor (TrkA) immunohistochemistry in the basal forebrain in L1 mice. Mean number of TrkA-ir interneurons in **(A)** the nucleus accumbens (NAc) and **(B)** striatum. Values expressed as mean ± S.D. *, p<0.05.

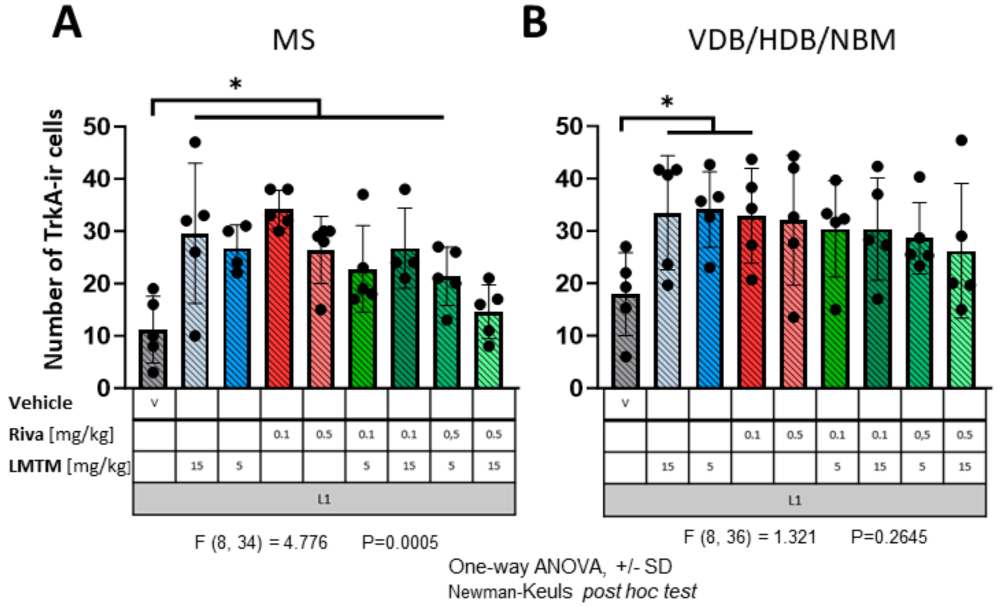

Figure S4. High-affinity nerve growth factor receptor (TrkA) immunohistochemistry in the basal forebrain in L1 mice. Mean number of TrkA-ir interneurons in **(A)** the medial septum and **(B)** total number of neurons in the vertical (VDB) and horizontal (HDB) limb of the diagonal band of Broca and the magnocellular basal nucleus-substantia innominata (NBM). Values expressed as mean ± S.D. *, p<0.05.

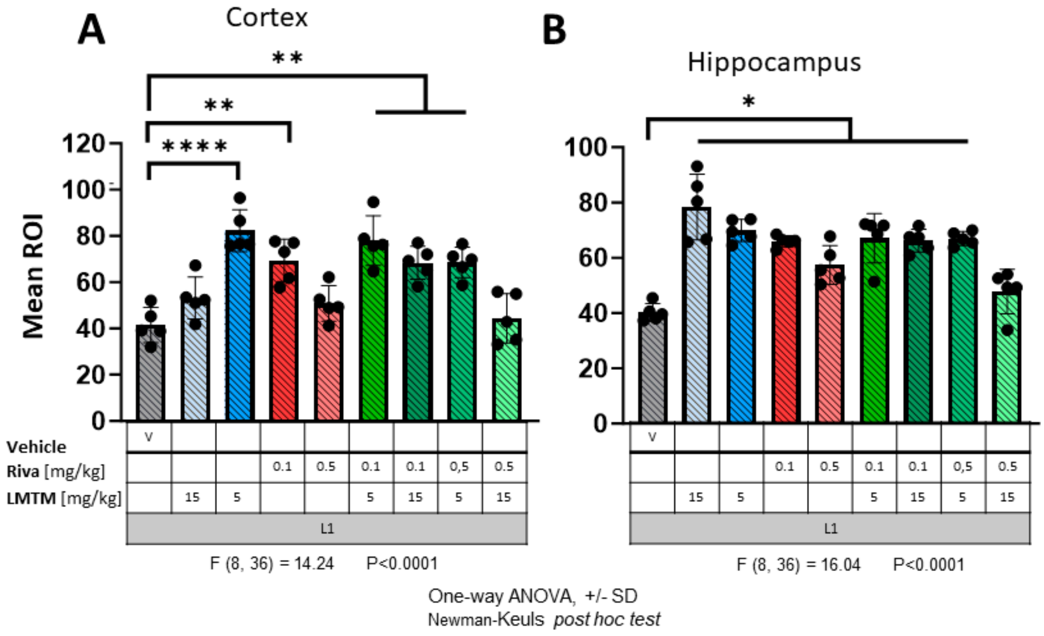

Figure S5. Vesicular acetylcholine transporter (VAChT) immunohistochemistry in L1 mice. Mean Relative Optical Density (Mean ROI) of VAChT staining in **(A)** motor cortex and **(B)** hippocampal CA3 field. Values expressed as mean ± S.D. *, p< 0.05; **, p< 0.01; ****, p<0.0001

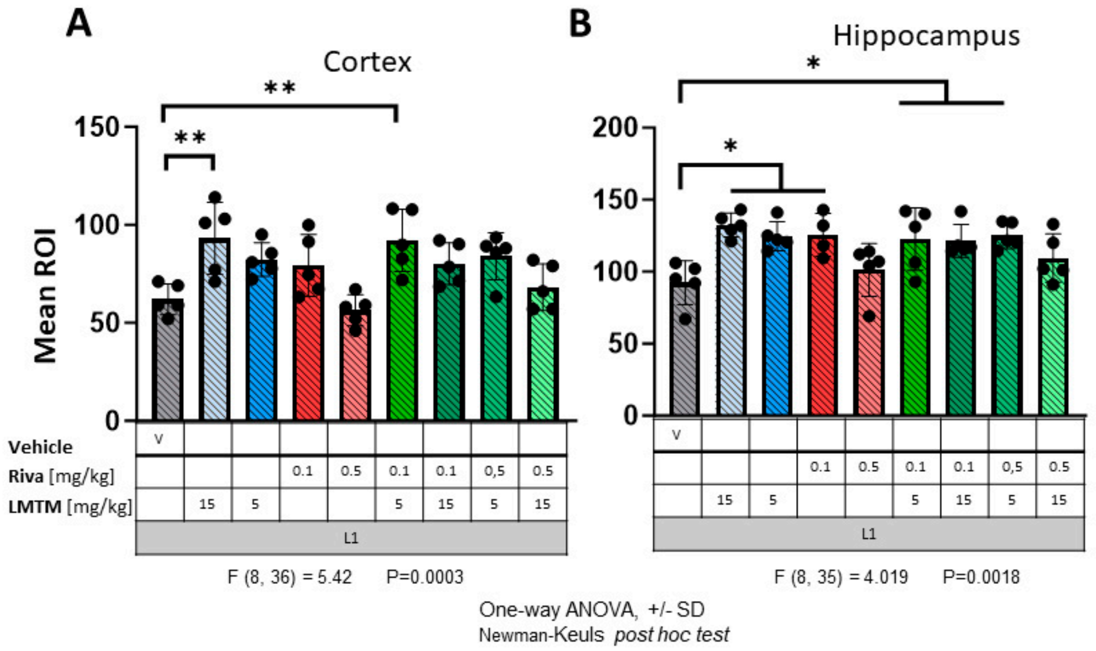

Figure S6. Acetylcholinesterase (AChE) in L1 mice. Mean Relative Optical Density (Mean ROI) of AChE staining in **(A)** motor cortex and **(B)** hippocampal CA3. Values expressed as mean ± S.D. *, p<0.05; **, p<0.01.

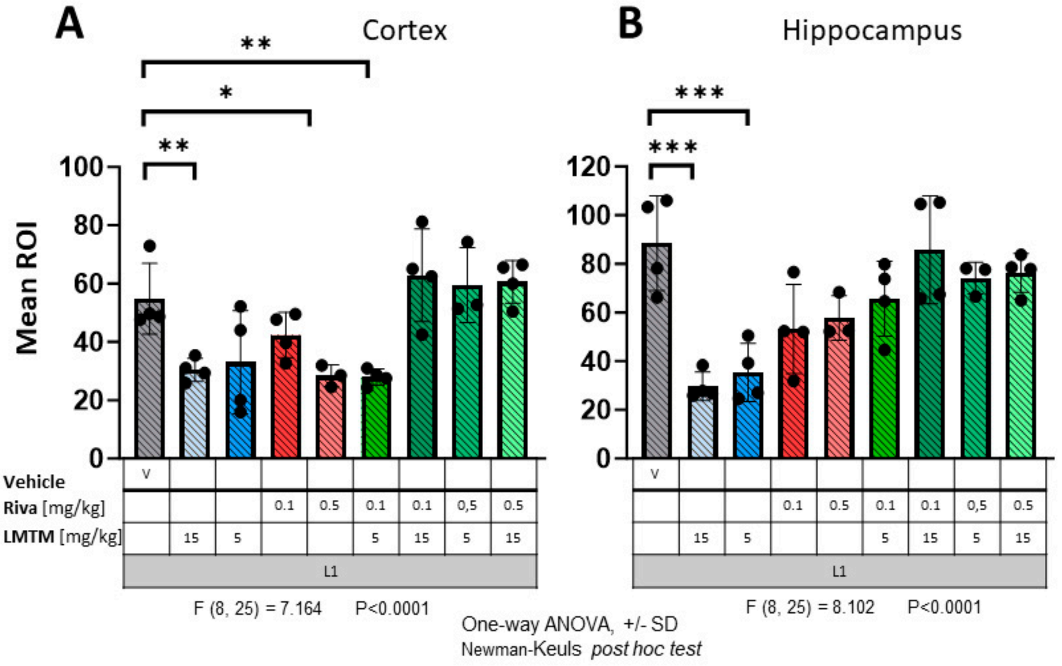

Figure S7. Tau immunohistochemistry, using S1D12, L1 mice. Mean Relative Optical Density (Mean ROI) of anti-tau staining in **(A)** motor cortex and **(B)** hippocampal CA3 field. Values expressed as mean ± S.D. *, p<0.05; **, p<0.01; ***p<0.001.
